# Nearly unbiased estimator of contemporary *N_e_*/*N* based on kinship relationships

**DOI:** 10.1002/ece3.6421

**Published:** 2020-09-23

**Authors:** Tetsuya Akita

**Affiliations:** ^1^ Japan Fisheries Research and Education Agency National Research Institute of Fisheries Science Yokohama Japan

**Keywords:** close‐kin mark‐recapture, effective population size, overdispersed reproduction, sibship assignment

## Abstract

This study develops a nearly unbiased estimator of the ratio of the contemporary effective mother size to the census size in a population, as a proxy of the ratio of contemporary effective size (or effective breeding size) to census size (*N_e_*/*N* or *N_b_*/*N*). The proposed estimator is based on both known mother–offspring (MO) and maternal‐sibling (MS) relationships observed within the same cohort, in which sampled individuals in the cohort probably share MO relationships with sampled mothers. The rationale is that the frequency of MO and MS pairs contains information regarding the contemporary effective mother size and the (mature) census size, respectively. Therefore, the estimator can be obtained only from genetic data. We also evaluate the performance of the estimator by running an individual‐based model. The results of this study provide the following: (a) parameter range for satisfying the unbiasedness, and (b) guidance for sample sizes to ensure the required accuracy and precision, especially when the order of the ratio is available. Furthermore, the results demonstrate the usefulness of a sibship assignment method for genetic monitoring, providing insights for interpreting environmental and/or anthropological factors fluctuating *N_e_*/*N* (or *N_b_*/*N*), especially in the context of conservation biology and wildlife management.

## Graphical abstract

We developed a nearly unbiased estimator of the ratio of the contemporary effective mother size to the census size in a population, as a proxy of *N_e_*/*N*. The proposed estimator is based on a known mother–offspring (MO) relationship and on a known maternal‐sibling (MS) relationship found within the same cohort, in which sampled individuals in the cohort potentially share MO relationships with sampled mothers.

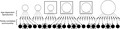

## 1 INTRODUCTION

The estimation of the ratio of the contemporary effective population size to the census size (*N_e_*/*N*) has attracted much research attention for providing information about a current population, especially in the context of conservation biology and wildlife management (Frankham, Bradshaw, & Brook, 2014; Palstra & Fraser, 2012). Small *N_e_*/*N* demonstrates large variance in reproductive success (Akita, 2020; Wang, Santiago, & Caballero, 2016; Waples, 2016), resulting from the variance of reproductive potential (e.g., the big old fat fecund female fish hypothesis; Hixon, Johnson, & Sogard, 2014) or from the situation in which only some families successfully reproduce (referred to as the “Sweepstakes reproductive success” hypothesis, Hedgecock & Pudovkin, 2011). Moreover, if *N_e_*/*N* is invariant across years, then *N_e_* may behave like an index of *N*, and vice versa (Luikart, Ryman, Tallmon, Schwartz, & Allendorf, 2010). However, if *N_e_*/*N* fluctuates across years, the trends can clarify the interpretation of environmental and/or anthropological factors, causing the variance of reproductive potential, family‐correlated survivorship, or fluctuating population dynamics. Besides, low precision and/or large bias for estimating *N_e_*/*N *may lead to a wrong interpretation of the population (Tallmon, Waples, Gregovich, & Schwartz, 2012).

The estimation of *N_e_*/*N *has been performed by utilizing the estimated values of contemporary effective population size (*N_e_*) and census size (*N*), unless complete pedigree data and/or full census data are available. Additionally, there are numerous methods for estimating *N_e_* from genetic markers (Wang et al., 2016, and the references contained therein). There are also numerous methods for estimating *N*, such as a mark‐recapture method or population dynamics modeling with survey data (e.g., Kéry & Schaub 2011; Methot & Wetzel, 2013; Quinn & Deriso, 1999; Seber, 2002). It is known that there are large variations in both estimators; thus, their combination (i.e., the estimator of *N_e_*/*N*) also shows large variation (Marandel et al., 2018; Palstra & Fraser, 2012). There is currently a little theoretical foundation for the estimator of *N_e_*/*N*, indicating no guidance for a sample size to ensure the required accuracy and precision.

Close‐kin mark‐recapture (CKMR) is a recently developed method for estimating *N* that utilizes the information about kinship in a sample. This was possible owing to the recent advances in genetic methods for kinship determination (Bravington, Grewe, & Davies, 2016; Bravington, Skaug, & Anderson, 2016; Hillary et al., 2018; Skaug, 2017), although similar methods have been proposed in the beginning of the 21st century (Nielsen, Mattila, Clapham, & Palsbøll, 2001; Pearse, Eckerman, Janzen, & Avise, 2001; Skaug, 2001). Besides, the rationale is that the presence of a kinship pair in the sample is analogous to the recapture of a marked individual in mark‐recapture. Kinship pairs in the sample are less likely to be observed in larger populations; thus, the number of kinship pairs may reflect *N*. While the original CKMR is designed to estimate adult abundance (i.e., *N*), the monitoring data for CKMR also produce the estimator of *N_e_* by detecting half‐sibling (HS) pairs within the same cohort (Akita, 2020). This kinship‐oriented estimation of *N_e_* was presented in the context of the sibship assignment method (Wang, 2009) and is expected to provide a much more accurate estimator as kinship determination becomes more accurate.

In this study, we propose a new method for estimating the ratio of contemporary effective mother size to the census size ( $N_{e,m}/N_{m}$) in a population, as a proxy of $N_{e}/N$. Assuming that kinships are genetically detected without any error, this method is based on the numbers of maternal‐sibling (MS) and mother–offspring (MO) pairs in a sample. Sampling is completed at a single breeding time; sampling offspring within the same cohort and mothers probably shares MO relationship with sampled offspring. Our model provides a nearly unbiased estimator of $N_{e,m}/N_{m}$ that explicitly incorporates two types of overdispersed reproduction (i.e., parental and nonparental variations; Akita, 2020), making it possible to target a species that shows iteroparity (i.e., multiple reproductive cycles during the lifetime) and/or sweepstakes reproductive success. This estimator applying an iteroparous species corresponds to the estimator of the ratio of contemporary effective breeding mother size to the census size, $N_{b,m}/N_{m}$. First, we explain the modeling assumption and sampling scheme. Then, we analytically determine (nearly) the unbiased estimators of $N_{e,m}$, $1/N_{m}$, and $N_{e,m}/N_{m}$, which are based on the numbers of MS and/or MO pairs. Finally, by running an individual‐based model, we investigate the performance of the estimator and provide a guide for a sample size. It is noteworthy that our modeling framework can be applied to diverse animal species. However, the description of the model focuses on fish species, which are presently the best candidate target of our proposed method.

## 2 THEORY

In this section, we present the theoretical foundation for estimating $N_{e,m}/N_{m}$. The estimator is based on previous studies that provide the estimator of $N_{e,m}$ (Akita, 2020) and $1/N_{m}$ (Akita, 2018) The main contribution of this paper is formulation of the estimator of $N_{e,m}/N_{m}$, presented in Equation 9. The main symbols used in the current paper are summarized in Table 1.

**Table 1:** The list of mathematical symbols employed in the main text

*n_M_*	Sample number of mother
*n_O_*	Sample number of offspring
*N*_m_	Number of mothers in the population when sampled offspring are born
*N*_e,m_	Effective number of mothers in the population
*N*_b,m_	Effective breeding number of mothers in the population
ϕ	Overdispersion parameter under negative binomial reproduction
$\lambda_{i}$	Expected number of surviving offspring of mother i at sampling
$f\left(\lambda \right)$	Frequency of λ for all mothers.
c	Combined effect of deviation from the Poisson ( $=\left(1+\phi^{-1}\right)E{\left[\lambda^{2}]/E[\lambda \right]}^{2}$)
$k_{i}$	Number of surviving offspring born to mother i
$H_{\text{MO}}$	Number of mother–offspring pairs observed in samples
$H_{\text{MS}}$	Number of maternal‐sibling pairs observed in samples
$\pi_{\text{MO}}$	Probability that a randomly selected pair (mother and offspring) shares a mother–offspring relationship
$\pi_{\text{MS}}$	Probability that a randomly selected pair (two offspring) shares a maternal‐sibling relationship
b	Bias of $\hat{N_{e,m}/N_{m}}$

### 2.1 Hypothetical population

**Figure 1:**
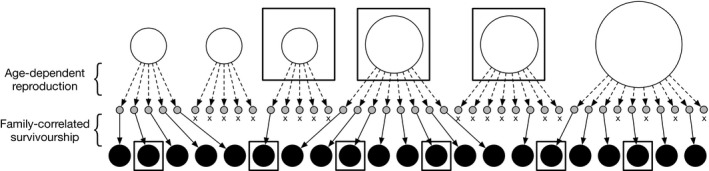
Example of relationships between mothers and their offspring number. The open, gray, and filled circles represent mothers, their eggs, and their offspring, respectively. The area of an open circle indicates the degree of reproductive potential of each mother (i.e., $\lambda_{i}$). The dotted and thin arrows denote mother–egg and egg–offspring relationships, respectively. The symbol × denotes a failure to survive at sampling. Sampled individuals are denoted with squares

We suppose that there is a hypothetical population comprising $N_{m}$ mothers and there is also no population subdivision or spatial structure. In this study, a mature female is called a mother even if she does not produce offspring. For mathematical tractability, we assume that only one spawning ground exists in which the mothers remain for the entire spawning season. Following (Akita, 2020), our modeling framework employs two types of overdispersed reproduction: parental and nonparental variations. Thus, the former indicates a variation caused by the mother's covariates, such as age, weight, and residence time on the spawning ground, while the latter indicates a variation caused by nonrandom stochastic events during a series of reproductive episodes, which are independent of the mother's covariates, such as family‐correlated survivorship or the mating behavior effects (e.g., competition for males/females and correlation between mating opportunities of the mother and the number of her offspring). Figure 1 illustrates a schematic diagram of the effects of parental and nonparental variations exemplified by age‐dependent reproduction and family‐correlated survival on kinship relationships in a population. Detailed definitions of parental and nonparental variations are stated in (Akita, 2020). Appendix 1 provides the theoretical foundation of both parental and nonparental variations in reproduction.

### 2.2 Sampling

To obtain the estimator of $N_{e,m}/N_{m}$, we utilize the number of MS and MO pairs observed in a sample. For the mathematical tractability, only one reproductive season is targeted for sampling. Thus, the sampling method does not affect our modeling framework whether it is invasive or noninvasive. In the population, $n_{M}$ mothers are randomly sampled immediately after the end of the reproductive season. Additionally, in the population, $n_{O}$ offspring are also randomly sampled. The numbers of MS and MO pairs are determined by pairwise comparison of all the sample individuals ( ${\;}_{n_{O}}C_{2}$ and $n_{M}n_{O}$ comparisons, respectively). As depicted in Figure 1, six offspring and three mothers are sampled and two MS and three MO pairs are observed. In our modeling framework, if an MS pair also shares a paternal‐sibling (PS) relationship, we count it as an MS pair and ignore the existing full‐sibling (FS) relationship.

### 2.3 Linking *N_e_*/*N*_m_ to kinship relationships

In this subsection, we provide the theoretical foundation for understanding how $N_{e,m}/N_{m}$ is associated with kinship relationships in a population, based on work presented in previous studies. The rationale is that the observed number of MS and MO pairs has information about *N*_e,m_ and *N*_m_, respectively, as noted below.

First, we consider the relationship between the number of MS pairs and $N_{e,m}$. (Akita, 2020) defined the contemporary effective mother size as follows:

(1) $$N_{e,m}=\frac{1}{\pi_{\text{MS}}},$$

where $\pi_{\text{MS}}$ denotes the probability that two offspring share an MS relationship with an arbitrary mother. This definition is related to the inbreeding effective population size (Nordborg & Krone, 2002; Wang, 2009). When sampling from a single cohort in a population with overlapping generations, the effective mother size in our definition corresponds to the effective breeding mother size per breeding‐time unit (e.g., year), which produces the single cohort and is denoted by $N_{b,m}$ (Waples, 1991). Hereafter, the description of the model focuses on species with discrete generations; thus, we use $N_{e,m}$ to denote the contemporary effective mother size, although $N_{b,m}$ is the appropriate notation in the left‐hand side of Equation 1 when the target species is iteroparous with overlapping generations, as exemplified in Appendix 2.

Given the total mother number and the degree of overdisepered reproduction in the population, (Akita, 2020) derived $\pi_{\text{MS}}$, which is approximately given by

(2) $$\pi_{MS}\approx \frac{c}{N_{m}+c-1},$$

where c indicates combined effect of both parental and nonparental variations and satisfies ≥1. Without either parental or nonparental variation (i.e., c→1, see details in Appendix 3), $\pi_{\text{MS}}$ converges to $1/N_{m}$, corresponding to the Poisson variance in reproduction for all mothers. The mathematical description of c is briefly summarized in Appendix 3.

Next, we consider the relationship between the number of MO pairs and $N_{m}$. It is natural to consider that the probability of a randomly sampled mother and her offspring sharing an MO relationship (denoted by $\pi_{\text{MO}}$) can be associated with the total mother number, given by

(3) $$\pi_{\text{MO}}=\frac{1}{N_{m}}.$$

It is noteworthy that $\pi_{\text{MO}}$ is independent of the distribution of the offspring number (Akita, 2018).

Together with Equations 1 and 3, we finally obtain the ratio of the effective mother size to census size as follows:

(4) $$\frac{N_{e,m}}{N_{m}}=\frac{\pi_{\text{MO}}}{\pi_{\text{MS}}},$$

indicating that $N_{e,m}/N_{m}$ is associated with kinship relationships (i.e., MS and MO) in a population. In other words, when $1/\pi_{\text{MS}}$ and $\pi_{\text{MO}}$ is estimated from observed MS and MO pairs, respectively, the ratio can also be estimated. Meanwhile, (Akita, 2020) obtained an alternative formulation of the ratio using Equations 1 and 2:

(5) $$\begin{array}{cc} \frac{N_{e,m}}{N_{m}} & =\frac{1}{\pi_{\text{MS}}}\frac{1}{N_{m}}\\ & \approx \frac{1}{c}, \end{array}$$

where $N_{m}\gg 1$ is assumed for approximation. This theoretical connection indicates that estimating $N_{e,m}/N_{m}$ corresponds to estimating 1/c.

### 2.4 Estimator of *N*_e,m_/*N*

This subsection proposes the estimator of $N_{e,m}/N_{m}$ as follows:

(6) $$\hat{\left(\frac{N_{e,m}}{N_{m}}\right)}=\hat{N_{e,m}}\hat{\left(\frac{1}{N_{m}}\right)}.$$

A “hat” denotes the estimator of a variable in this study. The requisite condition that satisfies Equation 6 is independent of $\hat{N_{e,m}}$ and $\hat{1/N_{m}}$. This property will be shown later in this subsection. Akita (2020) derived the nearly unbiased estimator of $N_{e,m}$, which is given by

(7) $$\begin{array}{cc} & \hat{N_{e,m}}=\hat{\left(\frac{1}{\pi_{\text{MS}}}\right)}\\ & =\frac{\left(\begin{array}{c} n_{O}\\ 2 \end{array}\right)+1}{H_{\text{MS}}^{\text{obs}}+1}, \end{array}$$

where $H_{MS}^{obs}$ denotes the observed number of MS pairs in a sample. This estimator works well unless $n_{O}$ is very small, which is based on the idea that the observation of $1/\left(H_{\text{MS}}+1\right)$ approximately provides a linear estimator of $N_{e,m}$.

Next, we consider to estimate $1/N_{m}$ by estimating $\pi_{\text{MO}}$. By definition of $\pi_{\text{MO}}$, we can set its estimator by $H_{\text{MO}}^{\text{obs}}/\left(n_{M}n_{O}\right)$, where $H_{\text{MO}}^{\text{obs}}$ denotes the observed number of MO pairs in a sample. Thus, using Equation 3, the estimator can be determined as follows:

(8) $$\begin{array}{cc} & \hat{\left(\frac{1}{N_{m}}\right)}=\hat{\pi_{\text{MS}}}\\ & =\frac{H_{\text{MO}}^{\text{obs}}}{n_{M}n_{O}}. \end{array}$$

Equation 8 provides a linear estimator of $1/N_{m}$, thus this estimator also works well. Meanwhile, the inverse of the right‐hand side in Equation 8 is a standard (moment) estimator of $N_{m}$ in the context of CKMR (Bravington, Skaug, et al., 2016).

Finally, substituting $\hat{N_{e,m}}$ (Equation 7) and $\hat{1/N_{m}}$ (Equation 8) into Equation 6, we obtain the estimator of $N_{e,m}/N_{m}$ as follows:

(9) $$\begin{array}{c} \hat{\left(\frac{N_{e,m}}{N_{m}}\right)}=\frac{\left(\begin{array}{c} n_{O}\\ 2 \end{array}\right)+1}{H_{\text{MS}}^{\text{obs}}+1}\frac{H_{\text{MO}}^{\text{obs}}}{n_{M}n_{O}}. \end{array}$$

Let both $n_{M}$ and $n_{O}$ be given. We numerically confirmed that there is no correlation between $H_{\text{MO}}^{\text{obs}}$ and $H_{\text{MS}}^{\text{obs}}$ (results are not shown). To intuitively explain this independency, we consider three mothers ( i=1,2,3) and their offspring, and assume that $\left(k_{1},k_{2},k_{3}\right)=\left(3,1,1\right)$ and $\left(n_{M},n_{O}\right)=\left(1,3\right)$. When the three offspring born to the first mother are sampled (i.e., $H_{\text{MS}}^{\text{obs}}=3$), the expected number of MO relationship is one ( =1/3×3+1/3×0+1/3×0). Meanwhile, when an offspring is sampled from each mother's offspring (i.e., $H_{\text{MS}}^{\text{obs}}=0$), the expected number of MO relationship is also one ( =1/3×1+1/3×1+1/3×1). Therefore, we conclude that both $\hat{N_{e,m}}$ and $\hat{1/N_{m}}$ are independent, and $\hat{N_{e,m}/N_{m}}$ is expected to work well (see details in the Section 3).

The bias of $\hat{N_{e,m}/N_{m}}$ is defined by b, which is approximately given by (see Appendix 5 for the derivation).

(10) $$\begin{array}{c} b=E\left[\hat{\left(\frac{N_{e,m}}{N_{m}}\right)}\right]-\left(\frac{N_{e,m}}{N_{m}}\right)\\ \approx -\left(\frac{N_{e,m}}{N_{m}}\right){\left(1-\frac{1}{N_{e,m}}\right)}^{\left(\begin{array}{c} n_{O}\\ 2 \end{array}\right)+1}. \end{array}$$

It is noteworthy that $\hat{N_{e,m}/N_{m}}$ is downwardly biased, especially when $n_{O}$ is very small. However, this bias may be ignored for a wide range of parameters (see details in the Section 3). Theoretically, b asymptotically converges to zero as $n_{O}$ increases, making $\hat{N_{e,m}/N_{m}}$ a nearly unbiased estimator. Moreover, as demonstrated in the Section 3, it is observed that an extremely skewed reproduction breaks down the unbiasedness (e.g., in the case that *c* = 20 and 100 in the results).

### 2.5 Individual‐based model

We developed an individual‐based model that tracks kinship relationships to evaluate the estimator's performance (e.g., $\hat{N_{e,m}/N_{m}}$). The population structure was assumed to be identical to that in the development of the estimators. In addition, the population comprised mothers and their offspring, and it was assumed to follow a Poisson or negative binomial reproduction (the degree of skewness due to the nonparental variation is controlled by a parameter ϕ; see Appendix 2 for details). The expected number of the surviving offspring of a mother (denoted by λ) followed the density distribution $f\left(\lambda \right)$ (which is involved to the parental variation; see Appendix 2 for details). We calculated overdispersion parameter ( c) from ϕ and $f\left(\lambda \right)$, as well as confirmed numerically that the value of c gives the same statistics of the estimators even if the combination of ϕ and $f\left(\lambda \right)$ differs (results are not shown). Therefore, each offspring retained the mother's ID, making it possible to trace an MS and MO relationship.

Let a parameter set ( $n_{O}$, $n_{M}$, $N_{m}$, ϕ, and parameters that determine $f\left(\lambda \right)$) be given. We simulated a population history in which $N_{m}$ mothers generated offspring; this process was repeated 100 times. The sampling process for each history was repeated 10,000 times, acquiring 1,000,000 data points that were utilized to construct the distribution of the estimators for each parameter set. Furthermore, true value of $N_{e,m}$ was calculated from $N_{m}$ and c (Equations 1 and 2).

## 3 RESULTS

We numerically evaluated the performance of $\hat{N_{e,m}/N_{m}}$ for the case in which the number of mothers, $N_{m}$, and the combined effect of deviation from the Poisson, c, were unknown. We changed the parameter values for $N_{m}$ ( $10^{3}$ and $10^{4}$) and c (1, 10, 20, and 100). In addition, based on a given parameter set ( $N_{m}$ and c), we mainly addressed the number of samples ( $n_{M}$ and $n_{O}$) required to obtain adequate accuracy and precision. In this study, we evaluated the performance of $\hat{N_{e,m}/N_{m}}$ for specific ranges of the sample sizes (50–200 when $N_{m}=10^{3}$, and 200–1,000 when $N_{m}=10^{4}$). Meanwhile, other estimators (i.e., $\hat{N_{e,m}}$ and $\hat{1/N_{m}}$) are also evaluated and provided in Supporting Information.

**Figure 2:**
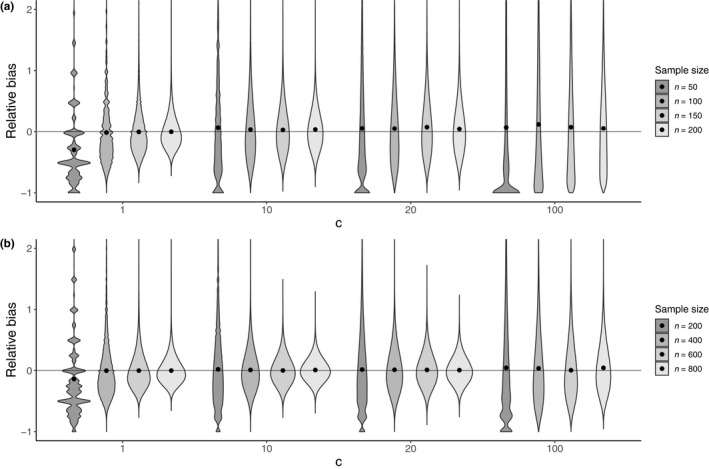
Violin plots showing the distribution of relative bias in our estimator (Equation 9) for various values of c and sample size. Filled circles represent the mean values. Sample sizes for mothers and offspring are identical (i.e., $n=n_{M}=n_{O}$) and indicated in the legend. For the demonstration purposes, the distribution is truncated, although the mean values are calculated including the truncated values. (a) $N_{m}=1,000$, (b) $N_{m}=10,000$

First, we evaluated the accuracy of estimators based on their relative bias calculated by applying the individual‐based model, which is defined as follows: “averaged estimator ‐ true value/true value.” For a given combination of $N_{m}$ and c, the value of the relative bias of $\hat{N_{e,m}/N_{m}}$ is represented on a violin plot for limiting cases where the sample number of mothers and offspring is same (i.e., $n_{M}=n_{O}$), as depicted in Figure 2. Meanwhile, detailed results of the relative bias are represented on a heatmap as a function of $n_{M}$ and $n_{O}$ (see Figure S1 in Supporting Information). For most of the investigated parameter sets, we observed that their relative bias is less than 10%. As expected, the relative bias is not affected by $n_{M}$ since $\hat{1/N_{m}}$ is exactly an unbiased estimator of $1/N_{m}$ (see Equation A11 in Appendix 4 and also Figure S3 in Supporting Information). Meanwhile, $\hat{N_{e,m}}$ is downwardly biased when $n_{O}$ is relatively small to true $N_{e,m}$ (e.g., see Figure S2 for c=1 in Supporting Information), as presented in (Akita, 2020); thus, $\hat{N_{e,m}/N_{m}}$ is downwardly biased. Contrary to the theoretical prediction for the direction of the bias (Equation 10), relatively strong overdispersion results in an upwardly bias for $\hat{N_{e,m}/N_{m}}$ when $N_{m}$ is relatively small and c is relatively large (e.g., c=20 and 100 in Figure 2a). This inconsistency may be caused by the breakdown of the approximation for deriving $\hat{N_{e,m}}$ (equation S14 in Akita, 2020). Thus, as described in Equation 2, extremely large c results in a large variance of offspring number, generating a situation in which the behavior of random variable $H_{\text{MS}}$ far deviates from the binomial distribution.

Next, we evaluated the precision of estimators based on their coefficient of variation. As demonstrated in Figure S4 in Supporting Information, the value of the coefficient of variation of $\hat{N_{e,m}/N_{m}}$ is also represented on a heatmap as a function of $n_{M}$ and $n_{O}$; meanwhile, the violin plot (Figure 2) visually provides the degree of precision. For the investigated parameter sets, the degree of the coefficient of variation strongly depends on the sample sizes. As shown in Figures S5 and S6 in Supporting Information, the dependency results from the combined effects of variation of both $\hat{1/N_{m}}$ and $\hat{N_{e,m}}$. As c increases, it is noteworthy that the parameter space of sample sizes demonstrating large variation of $\hat{1/N_{m}}$ (e.g., CV>30%) expands; however, when c is small (e.g., c=1), relatively small $n_{O}$ results in large variation of $\hat{N_{e,m}}$ because of a relatively large $N_{e,m}$.

## 4 DISCUSSION

We theoretically developed a nearly unbiased estimator of the ratio of contemporary effective mother size to the census size ( $N_{e,m}/N_{m}$) in a population (Equation 9). The proposed estimator is based on known MO relationship and MS relationships observed within the same cohort, in which sampled individuals in the cohort probably share MO relationships with sampled mothers (Figure 1). The performance of the estimator (accuracy and precision) was quantitatively evaluated by running an individual‐based model (Figure 2; see also Figures S1–S6). Meanwhile, the bias is analytically provided (Equation 10). Our modeling framework utilizes two types of reproductive variations (Akita, 2020): variance of the average offspring number per mother (parental variation, denoted by $f\left(\lambda \right)$), and variance of the offspring number across mothers with the same reproductive potential (nonparental variation, denoted by ϕ). Additionally, these two effects result in a skewed distribution of offspring number and are summarized into one parameter ( c) in the framework. Thus, our estimator can be calculated from sample sizes of mother and offspring ( $n_{M}$ and $n_{O}$, respectively) and the observed numbers of MS and MO pairs ( $H_{\text{MS}}^{\text{obs}}$ and $H_{\text{MO}}^{\text{obs}}$, respectively), and it does not require other parameters. The rationale for this is the following: (a) the frequency of MS and MO pairs contains information about $N_{e,m}$ and *N_m_*, respectively; (b) the estimators of $N_{e,m}$ and $1/N_{m}$ are independently determined based on a pedigree structure in the population and sample sizes, generating the estimator of $N_{e,m}/N_{m}$ by multiplying both estimators ( $\hat{N_{e,m}}$ and $\hat{1/N_{m}}$). In this study, although $\hat{N_{e,m}/N_{m}}$ is considered as a proxy of $\hat{N_{e}/N}$, our theoretical results can easily be extended to the estimator of the ratio of contemporary effective father size to the census size if fathers are also sampled. The comparison of both ratios could clarify the underlying processes that differentiate between the sexes in the context of reproductive ecology.

The novelty of this study is that $\hat{N_{e,m}/N_{m}}$ can be obtained only from the genetic data, and there are numerous advantages in using the proposed estimator instead of separately estimating $N_{e}$ (via genetical method) and N (via nongenetical method). First, sampling and analyzing designs have become substantially simplified. Requirements for the proposed estimator are sampling of mothers and (potentially) their offspring in an appropriate time, and the extraction of their DNA that satisfies an adequate number of markers for kinship detection. In addition, both MO and MS pairs can be detected by a applying unified framework of genetic analyzes (there are many algorithms to detect kinship pairs from single nucleotide polymorphisms (SNPs) or short tandem repeats (STRs)), although an MS pair involves many more DNA markers (e.g., several thousands of SNPs are required for detection) than an MO pair (e.g., several hundreds of SNPs are required for detection). Second, our theoretical results guide sample sizes (*n_M_* and *n_O_*) to ensure the required accuracy and precision, especially if the order of the number of effective mothers is approximately known. This is due to the simple formulation of the estimator determined only by the observed values (Equation 9). Third, the proposed estimator directly reflects the amounts of $N_{e,m}$ and $N_{m}$ at the same timing (i.e., immediately after the end of the reproductive season), leading to a clear interpretation of the results, especially for genetic monitoring. For example, when the strong cohort is added to the spawning population in the beginning of the year, the estimator of *N_e_* without reflecting this addition may results in an inappropriate estimation of $N_{e}/N$ (details of the temporal scale relevant to estimated *N_e_* for each method were discussed in Wang et al., 2016).

Our modeling framework is presented by combining the context of the sibship assignment method (for estimating $N_{e,m}$) and the CKMR method (for estimating $1/N_{m}$), which defines a kinship‐oriented estimation of effective/census population size. Improvements to these methods directly contribute to the estimation of $N_{e,m}/N_{m}$. Furthermore, the original theory of the sibship assignment method requires HS and FS pairs but does not require a distinction between the MS and PS pairs (Wang, 2009). This is a significant advantage due to the difficulty of the distinction from genetic data. However, the limitation of using MS or PS pair enables us to employ a nearly unbiased estimator of $N_{e}$ for particular sex (Akita, 2020), which greatly improves the accuracy of the estimation of the $N_{e,m}$ in this study and thus that of $N_{e,m}/N_{m}$ (see figure E3 in Appendix 5). It is noteworthy that the estimator of 1/N is given by

(11) $$\hat{\left(\frac{1}{N}\right)}=\frac{H_{\text{PO}}^{\text{obs}}}{2n_{P}n_{O}},$$

where $n_{P}$ and $H_{\text{PO}}^{\text{obs}}$ denotes the sample size of the parent and the observed number of parent–offspring (PO) pairs in a sample, respectively (Bravington, Skaug, et al., 2016). The development of the unbiased estimator of $N_{e}$ without a distinction between MS and PS pairs that could provide an unbiased estimator of $N_{e}/N$ coupled with Equation 11, is a study for the future. Furthermore, using cross‐cohort HS pairs, the CKMR method also provides the estimator of N (Bravington, Skaug, et al., 2016) that does not require the sampling of the parent, which probably provides the estimator of $N_{e}/N$ only from unmatured samples. This perspective of the study will also be conducted in the future.

Finally, we note the advantage of partitioning variance in reproductive success into two components. As denoted in Equation A8 in Appendix 3, the combined effect of parental and nonparental variations is given by

(12) $$c=\left(\text{effect of nonparental variation}\right)\times \left(\text{effect of parental variation}\right).$$

Meanwhile, as denoted in Equation 5, the estimator of *N*_e,m_/*N_m_* provides the information for the left‐hand side of Equation 12. Thus, when we obtain parental variation information from the life‐history table or from other species, the estimator of *N*_e,m_/*N_m_* can also estimate the degree of nonparental variation. This procedure provides an insight into sweepstake reproduction or family‐correlated sampling of offspring (i.e., nonrandom sampling), although the theoretical formulation and its evaluation remain a task for future research. Alternatively, Waples et al. (2018) developed a genetical method for estimating $N_{e}/N$ and $N_{b}/N$ via estimating the degree of nonparental variation from fecundity data in southern bluefin tuna.

## CONFLICT OF INTEREST

The author declares no conflict of interest.

## AUTHOR CONTRIBUTION

**Tetsuya Akita:** Conceptualization (lead); Formal analysis (lead); Funding acquisition (lead); Investigation (lead); Methodology (lead); Project administration (lead); Resources (lead); Software (lead); Supervision (lead); Validation (lead); Visualization (lead); Writing‐original draft (lead); Writing‐review & editing (lead).

## Supporting information

Figures S1‐6

## Figures and Tables

**Figure A1:**
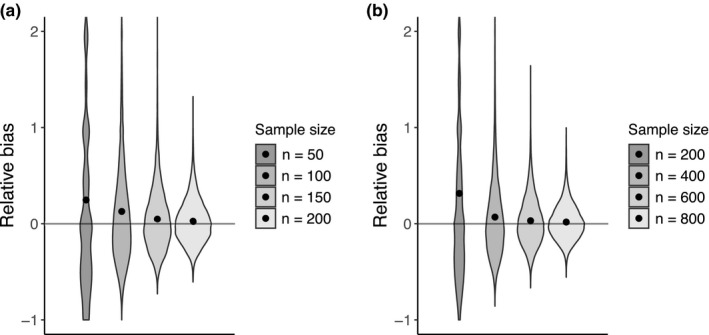
Violin plots showing the distribution of relative bias of $N_{e}/N$ (denoted in Equation A13) for various sample sizes. Filled circles represent the mean values. The sample numbers of parents and offspring are identical (i.e., $n=n_{P}=n_{O}$) and indicated in the legend. For demonstration purposes, the distribution is truncated, although the mean values are calculated including the truncated values. (a) N=2,000, (b) N=20,000

## Data Availability

No datasets were generated or analyzed in this study.

## APPENDIX 1

### Distribution of the number of surviving offspring per mother and its variance

Let $k_{i}$ denote the total number of surviving offspring from a mother i ( $i=1,2,\ldots ,N_{m}$) at time of sampling. Following (Akita, 2020) and giving the expected number of the surviving offspring per mother at time of sampling, $\lambda_{i}$ (>0), $k_{i}$ is assumed to follow a negative binomial distribution through a conventional parametrization:

(A1) $$Pr\left[k_{i}|\lambda_{i}\right]=\frac{\Gamma \left[k_{i}+\phi \right]}{k_{i}!\Gamma \left[\phi \right]}{\left(\frac{\lambda_{i}}{\phi +\lambda_{i}}\right)}^{k_{i}}{\left(\frac{\phi }{\phi +\lambda_{i}}\right)}^{\phi },$$

where ϕ (>0) denotes the overdispersion parameter describing the degree of nonparental variation. In the current work, ϕ is assumed to be constant across mothers, whereas the expected number of surviving offspring ( $\lambda_{i}$) varies across mothers. The mean and variance of this distribution are denoted by $\lambda_{i}$ and $\lambda_{i}+\lambda_{i}^{2}/\phi$, respectively. In the limit of infinite ϕ, this distribution becomes a Poisson distribution, which is given by $Pr\left[k_{i}|\lambda_{i}\right]=\lambda_{i}^{k_{i}}e^{-\lambda_{i}}/\left(k_{i}!\right)$. We assume $\lambda_{i}$ to be independent and identically distributed with the density function $f\left(\lambda \right)$, producing a parental variation. The shape of the density function is often complex, but may be described by information from the population, for example, the mother's weight composition in the population. The specific form of $f\left(\lambda \right)$ is given in Appendix 2 and is used for running an individual‐based model.

The variance of the offspring number can be given by

(A2) $$\begin{array}{cc} & V\left[k\right]=E\left[V[k|\lambda ]\right]+V\left[E[k|\lambda ]\right]\\ & \;=E\left[\lambda +\lambda^{2}/\phi \right]+V\left[\lambda \right]. \end{array}$$

## APPENDIX 2

### Probability density function and its moment of offspring number per mother

As stated in the main text, our modeling framework does not require the specific form of the density function of offspring number per mother (denoted by $f\left(\lambda \right)$); it only requires the ratio of the second moment to the squared first moment ( $E\left[\lambda^{2}\right]/E{\left[\lambda \right]}^{2}$) instead. However, the specific form is required for evaluating the theoretical results (i.e., calculating the moment or running the individual‐based model). Herein, we model an age‐structured fish population that serves as a representative example, demonstrating both parental and nonparental variations. The following contents are almost the same as those of (Akita, 2020) except for the parameter values that produce the setting *c* = 20 and 100.

Suppose that the mean fecundity of a mother depends on her age. Let $\lambda_{a}$ denote the mean fecundity, which is a function of age (denoted by a). The moment can be defined as $E\left[\lambda^{m}\right]=\sum_{a=0}^{a_{\max}}\lambda_{a}^{m}h_{\text{mat}}\left(a\right)$, where $h_{\text{mat}}\left(a\right)$ is the frequency of mature mothers at a given age, and *a*_max_ denotes the maximum age. Thus, we can numerically obtain the moment by applying $\lambda_{a}$ and $h_{\text{mat}}\left(a\right)$.

For marine species with a type‐III survivorship curve, it is generally assumed that individual fecundity is proportional to weight. By utilizing the von Bertalanffy growth equation for body weight, $\lambda_{a}$ is explicitly defined as a function of age as follows:

(A3) $$\lambda_{a}\propto {\left(1-exp\left[-\kappa \left(a-a_{0}\right)\right]\right)}^{\beta },$$

where κ, $a_{0}$, and β are conventionally used parameters in the von Bertalanffy equation, and they denote the growth rate, the adjuster of the equation for the initial size of the animal, and the allometric growth parameter, respectively. To obtain a specific value of λ, a coefficient value of 10 multiplied by the right‐hand side of Equation A3 was used when running the individual‐based model.

The frequency of mature mothers at a given age can be given as the following:

(A4) $$h_{mat}\left(a\right)\propto h\left(a\right)Q\left(a\right),$$

satisfying $\sum_{a=0}^{a_{\max}}h_{\text{mat}}\left(a\right)=1$, where $h\left(a\right)$ and $Q\left(a\right)$ denote the frequency and maturity at a given age, respectively. Although $f\left(a\right)$ is affected by historical population dynamics and age‐dependent survival, for simplicity, the mortality rate is assumed to be constant (i.e., age independent):

(A5) $$\begin{array}{c} h\left(a\right)\propto \left\{\begin{array}{c} \begin{array}{cc} S^{a} & \text{if}\;a<a_{\max}\\ 0 & \text{if}\;a=a_{\max} \end{array} \end{array}\right., \end{array}$$

where *S *denotes a survival probability. The maturity at age (*Q*(*a*)) is assumed to be a knife‐edge function, which is given by

(A6) $$\begin{array}{c} Q\left(a\right)=\left\{\begin{array}{c} \begin{array}{cc} 1 & \text{if}\;a\geq a_{\text{mat}}\\ 0 & \text{otherwise} \end{array} \end{array}\right., \end{array}$$

where *a*_mat_ denotes the mature age.

To calculate $E{\left[\lambda^{2}]/E[\lambda \right]}^{2}$, the required parameter set is $\left(a_{\max},\kappa ,a_{0},\beta ,S,a_{\text{mat}}\right)$. In this study, for the purpose of representation, we fixed the values of several parameters as follows: $a_{max}=20$, κ=0.3, $a_{0}=0$, S=0.5 and $a_{\text{mat}}=0$. In addition, we selected parameter value c (defined in Equation A8 in the main text) to be 1, 10, 20, and 100 for comparison with the results in the main text that are derived from the parameter set $\left(\phi ,\beta \right)=\left(1000,0.0009\right)$, $\left(0.1302,0.9\right)$, $\left(0.06111,0.9\right)$, and $\left(0.01165,0.9\right)$, respectively.

Finally, we provide specific forms of $f\left(\lambda \right)$; thus, when $\lambda_{a}$ and $h_{\text{mat}}\left(a\right)$ are obtained, $f\left(\lambda \right)$ is given by

(A7) $$\begin{array}{c} f\left(\lambda \right)=\left\{\begin{array}{c} \begin{array}{cc} h_{\text{mat}}\left(a\right) & \text{if}\;\lambda =\lambda_{a}\\ 0 & \text{otherwise} \end{array} \end{array}\right.. \end{array}$$

## APPENDIX 3

### Combined effect of parental and nonparental variations

Following (Akita, 2020), combined effect of parental and nonparental variations can be partitioned into the two components, such as:

(A8) $$c=\left(1+\phi^{-1}\right)\times \left(\frac{E[\lambda^{2}]}{E{\left[\lambda \right]}^{2}}\right).$$

The first and second parentheses give the effect of nonparental variation and parental variation, respectively. When λ is constant across mothers, $E[\lambda^{2}]$ equals $E{\left[\lambda \right]}^{2}$ and c converges to $\left(1+\phi^{-1}\right)$; meanwhile, ϕ→∞, c converges to $E{\left[\lambda^{2}]/E[\lambda \right]}^{2}$. Without either parental or nonparental variation, c converges to 1 and $\pi_{MS}$ converges to $1/N_{m}$. In the main text, we refer to “overdispersion” as the skewed distribution of the offspring number resulting from this combined effect.

Using Equations A2 and A8, the variance of the offspring number can be expressed as

(A9) $$V\left[k\right]=E\left[\lambda \right]+E{\left[\lambda \right]}^{2}\left(c-1\right),$$

suggesting that the variance substantially increases with c.

## APPENDIX 4

**Derivation of the bias of** $\hat{N_{e,m}/N_{m}}$

For calculation of the bias of $\hat{N_{e,m}/N_{m}}$, we require an expectation of the estimator given by

(A10) $$E\left[\hat{\left(\frac{N_{e,m}}{N_{m}}\right)}\right]=COV\left[\hat{\left(\frac{1}{N_{m}}\right)},\hat{N_{e,m}}\right]+E\left[\hat{\left(\frac{1}{N_{m}}\right)}\right]E\left[\hat{N_{e,m}}\right].$$

As stated in the main text, both $\hat{N_{e,m}}$ and $\hat{1/N_{m}}$ are independent. Thus, the first term in the right‐hand side of Equation A10 can be ignored. The expectation of $\hat{1/N_{m}}$ is given by

(A11) $$\begin{array}{cc} & E\left[\hat{\left(\frac{1}{N_{m}}\right)}\right]=\frac{E\left[H_{MO}\right]}{n_{M}n_{O}}\\ & \;=\frac{1}{N_{m}}. \end{array}$$

From the first to the second line of Equation A11, we applied the relationship $\pi_{MO}=E\left[\pi_{MO}]=E[H_{MO}\right]/\left(n_{M}n_{O}\right)$ and Equation 3. Equation A11 indicates that $\hat{1/N_{m}}$ is the unbiased estimator. The expectation of $\hat{N_{e,m}}$ is given by

(A12) $$\begin{array}{c} E\left[\hat{N_{\text{e,m}}}\right]=N_{\text{e,m}}-N_{\text{e,m}}{\left(1-\frac{1}{N_{\text{e,m}}}\right)}^{\left(\begin{array}{c} n_{O}\\ 2 \end{array}\right)+1}, \end{array}$$

which is illustrated in Appendix 4 of (Akita, 2020). Together with these relationships, we can obtain the bias of $\hat{N_{e,m}/N_{m}}$ described in Equation 10.

## APPENDIX 5

### Individual‐based model with application of Wang's estimator

Our modeling framework estimates the ratio of contemporary effective mother size to census size (denoted by $N_{e,m}/N_{m}$), as a proxy for the ratio of contemporary effective size to census size (denoted by $N_{e}/N$). To compare the performance of our method with that of other methods for directly estimating $N_{e}/N$, we propose the estimator of $N_{e}/N$, given by

(A13) $$\begin{array}{cc} & \hat{\left(\frac{N_{e}}{N}\right)}=\hat{N_{e}}\hat{\left(\frac{1}{N}\right)}\\ & =\frac{4\left(\begin{array}{c} n_{O}\\ 2 \end{array}\right)}{H_{\text{HS}}^{\text{obs}}+H_{\text{FS}}^{\text{obs}}}\frac{H_{\text{PO}}^{\text{obs}}}{2n_{P}n_{O}}. \end{array}$$

$n_{P}$ and $n_{O}$ indicate the sample numbers of parents and offspring, respectively, and $H_{\text{HS}}^{\text{obs}}$, $H_{\text{FS}}^{\text{obs}}$, and $H_{\text{PO}}^{\text{obs}}$ indicate the observed number of half‐sibling, full‐sibling, and parent–offspring pairs, respectively. $\hat{N_{e}}$ is based on (Wang, 2009) assuming random mating and $\hat{1/N}$ is defined in Equation 11 in the main text.

We evaluated the estimator's performance on data simulated by running an individual‐based model under the Wright–Fisher process for a diploid population. In the current comparison, we did not consider the case of overdispersion (i.e., $N_{e}=N$). Sex ratio was fixed to 0.5 in both whole and sampled populations. Each parent retained the ID of its offspring, making it possible to trace HS, FS, and PO relationships. Given a parameter set ( N, $n_{O}$, and $n_{P}$), we backwardly simulated a population history in which mother–offspring and father–offspring relationships were randomly specified from $n_{O}$ offspring; this process was repeated 10,000 times, acquiring 10,000 data points that were used to construct the distribution of $\hat{N_{e}/N}$ for each parameter set. If neither HS nor FS pairs were found in a sample, we did not include that trial when constructing the distribution. For ease of comparison, we used the same sample size and population structure for both methods.

Figure A1 illustrates the distribution of the relative bias of $\hat{N_{e}/N}$ for limiting cases where parent and offspring sample numbers are identical (i.e., $n_{P}=n_{O}$). Our results indicate that the estimator is upwardly biased, particularly when sample size is small. Alternatively, our estimator shows a broader region such that unbiasedness is approximately achieved. For example, when n=100 and 150 in *N* = 2,000 (i.e.,* N_m_* = 1,000), the bias of $\hat{N_{e}/N}$ is clearly observed (Figure E1a); meanwhile, $N_{e,m}/N_{m}$ does not produce a bias in these conditions (Figure 2a).
