# Supplementary material for: Nearly unbiased estimator of contemporary Ne/N based on kinship relationships
Source: Ecol Evol. 2020 Sep 23;10(19):10343–52. doi: 10.1002/ece3.6421 (PMC7548192; doi:10.1002/ece3.6421)
Supplement: Supplementary file 1 — Figures S1‐6 [file ECE3-10-10343-s001.pdf]

Supporting Information

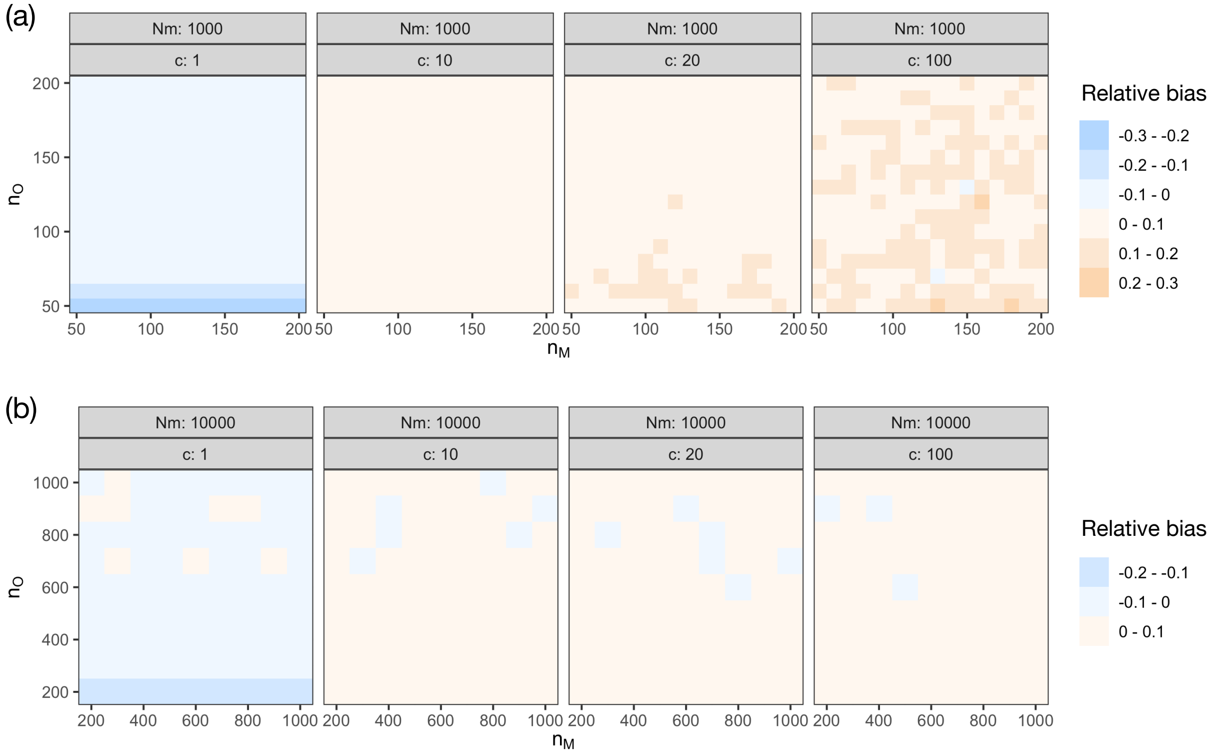

FIGURE S1 Heatmap showing the relative bias of $\widehat{N_{\text{e,m}}/N_{\text{m}}}$ as a function of both n_{M} and n_{O}. The value of the relative bias is indicated in the legend. The value of the combined effect of parental and nonparental variations increases from left to right ($c = 1, 10, 20$, and 100). (a) $N_{\text{m}} = 1{,}000$, (b) $N_{\text{m}} = 10{,}000$.

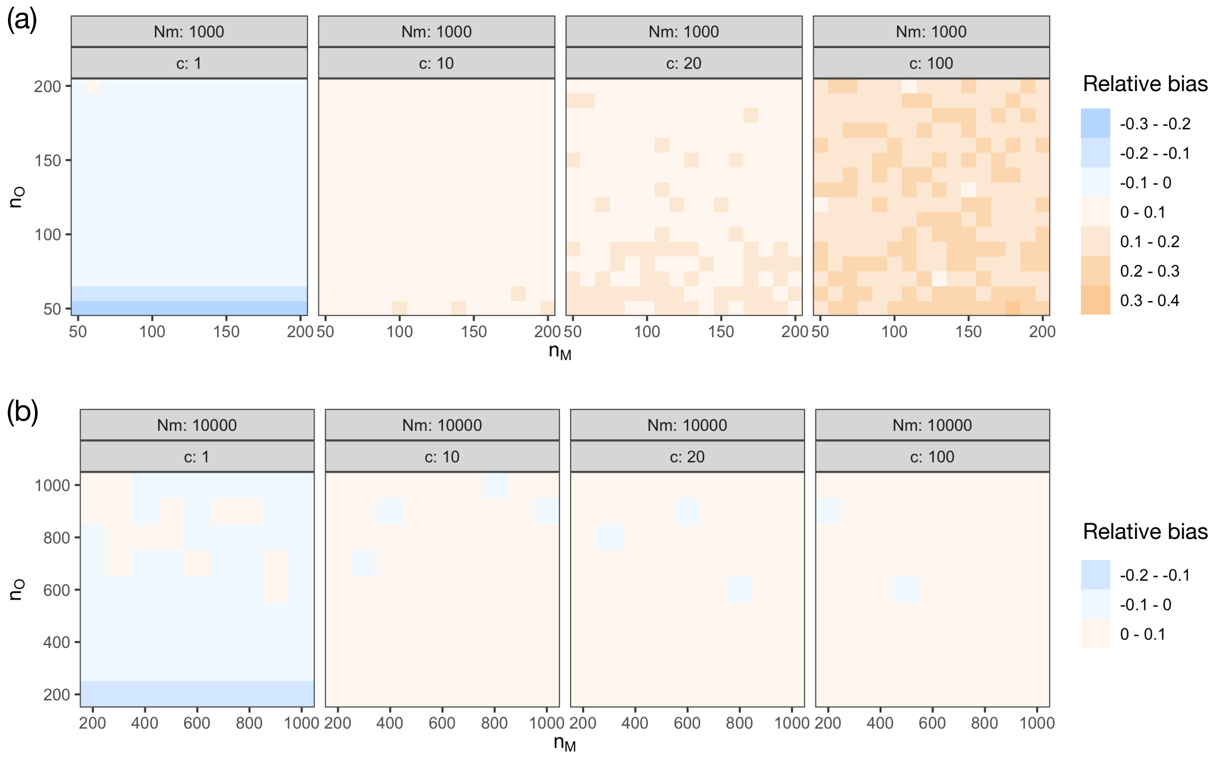

FIGURE S2 Heatmap showing the relative bias of $\widehat{N_{e,m}}$ as a function of both n_M and n_O. The value of the relative bias is indicated in the legend. The value of the combined effect of parental and nonparental variations increases from left to right ($c = 1, 10, 20$, and 100). (a) $N_m = 1{,}000$, (b) $N_m = 10{,}000$.

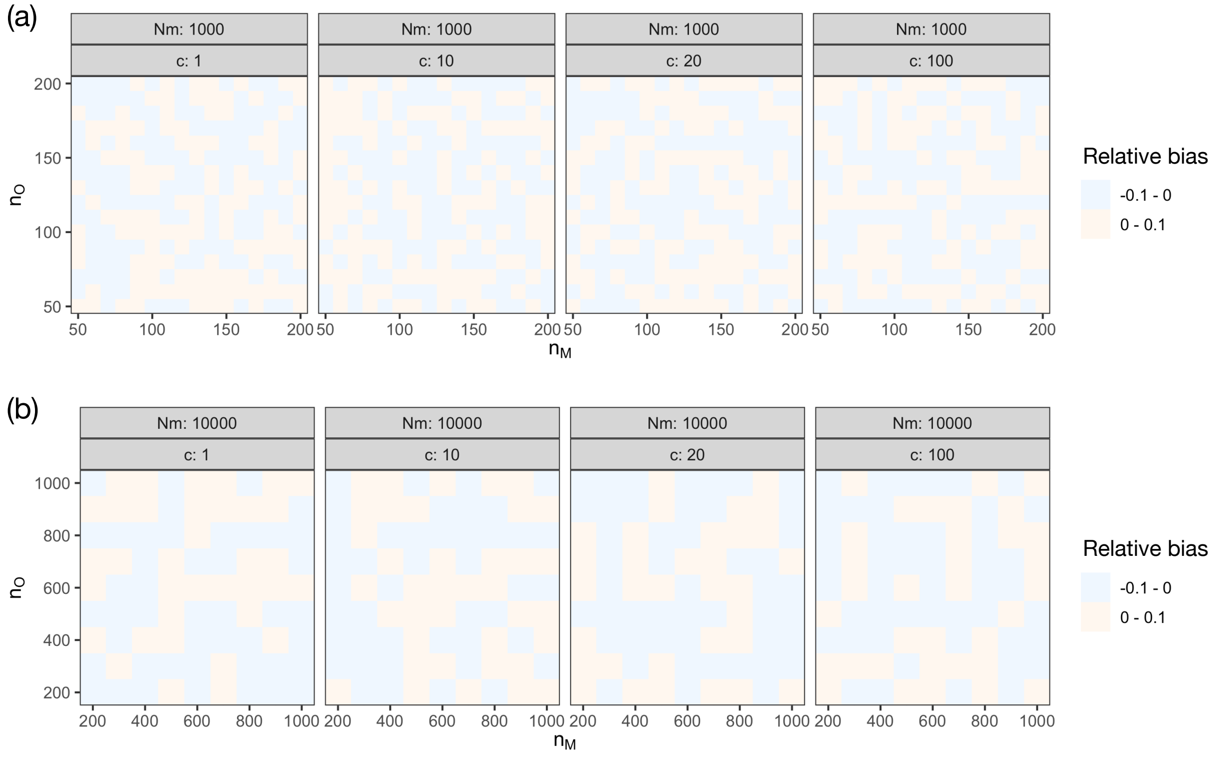

FIGURE S3 Heatmap showing the relative bias of $\widehat{1/N_{\mathrm{m}}}$ as a function of both n_{M} and n_{O}. The value of the relative bias is indicated in the legend. The value of the combined effect of parental and nonparental variations increases from left to right ($c = 1, 10, 20$, and 100). (a) $N_{\mathrm{m}} = 1{,}000$, (b) $N_{\mathrm{m}} = 10{,}000$.

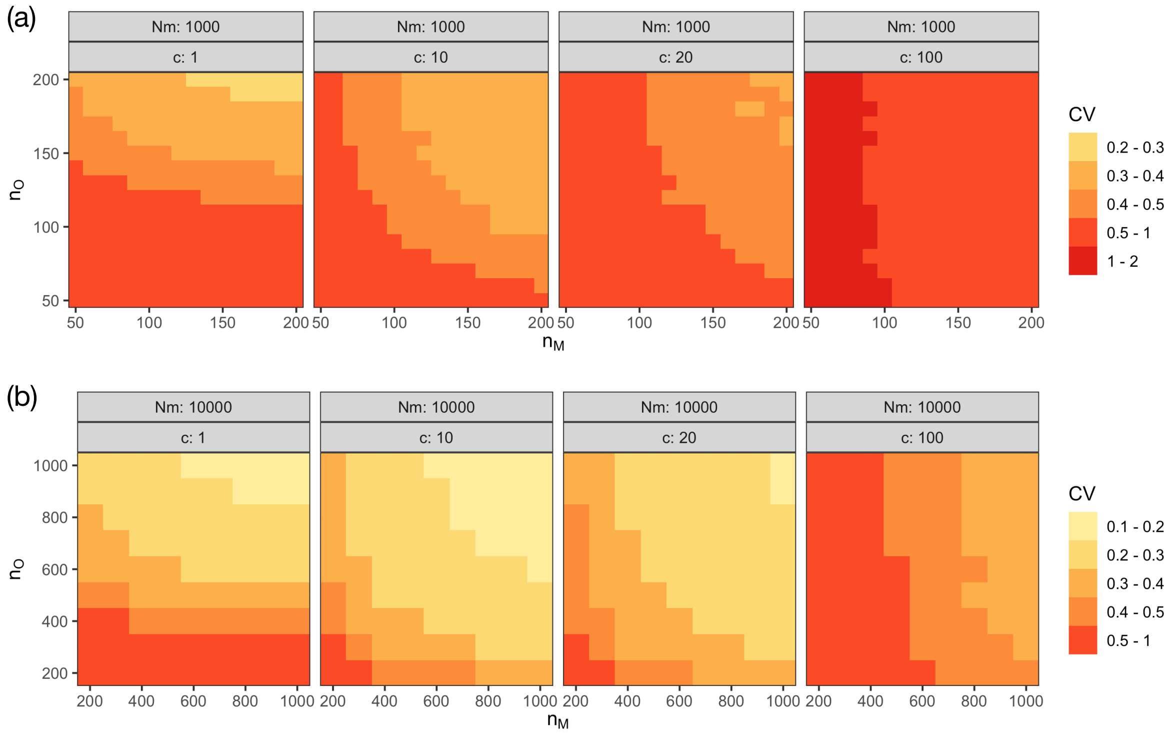

FIGURE S4 Heatmap showing the coefficient of variation of $\widehat{N_{\rm e,m}/N_{\rm m}}$ as a function of both $n_{\rm M}$ and $n_{\rm O}$. The value of the coefficient of variation is indicated in the legend. The value of the combined effect of parental and nonparental variations increases from left to right ($c = 1, 10, 20$, and 100). (a) $N_{\rm m} = 1{,}000$, (b) $N_{\rm m} = 10{,}000$.

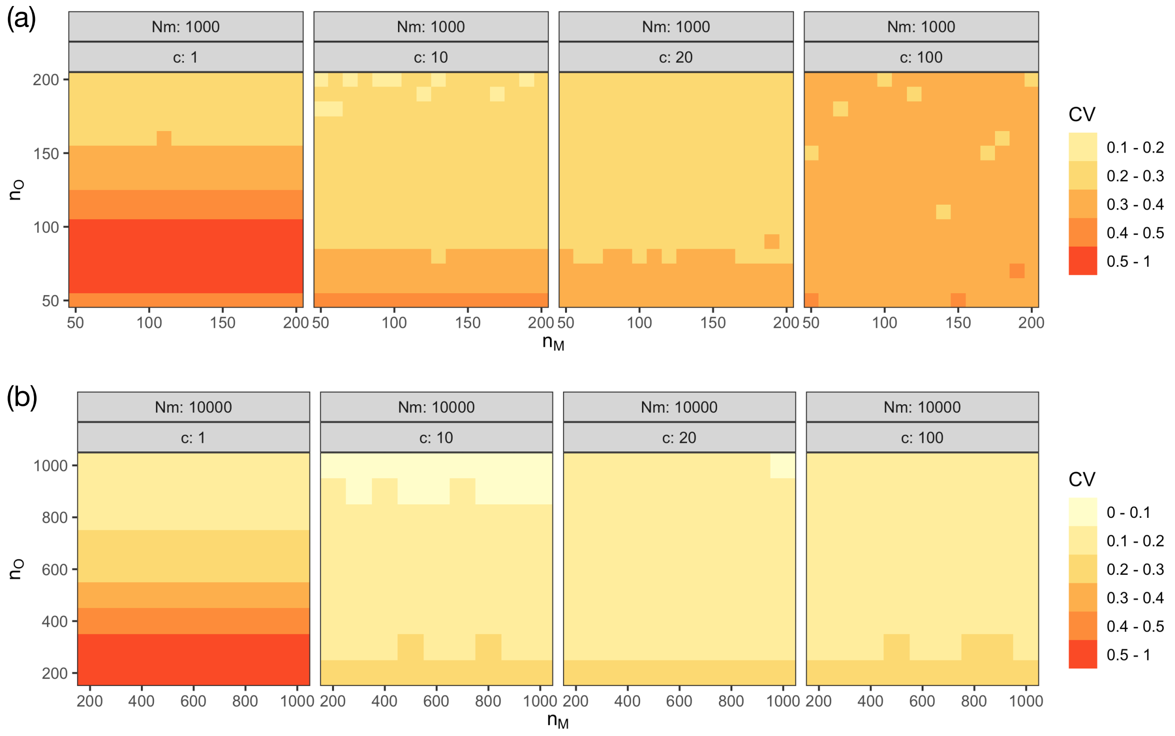

FIGURE S5 Heatmap showing the coefficient of variation of $\widehat{N_{e,m}}$ as a function of both n_M and n_O. The value of the coefficient of variation is indicated in the legend. The value of the combined effect of parental and nonparental variations increases from left to right ($c = 1, 10, 20$, and 100). (a) $N_m = 1{,}000$, (b) $N_m = 10{,}000$.

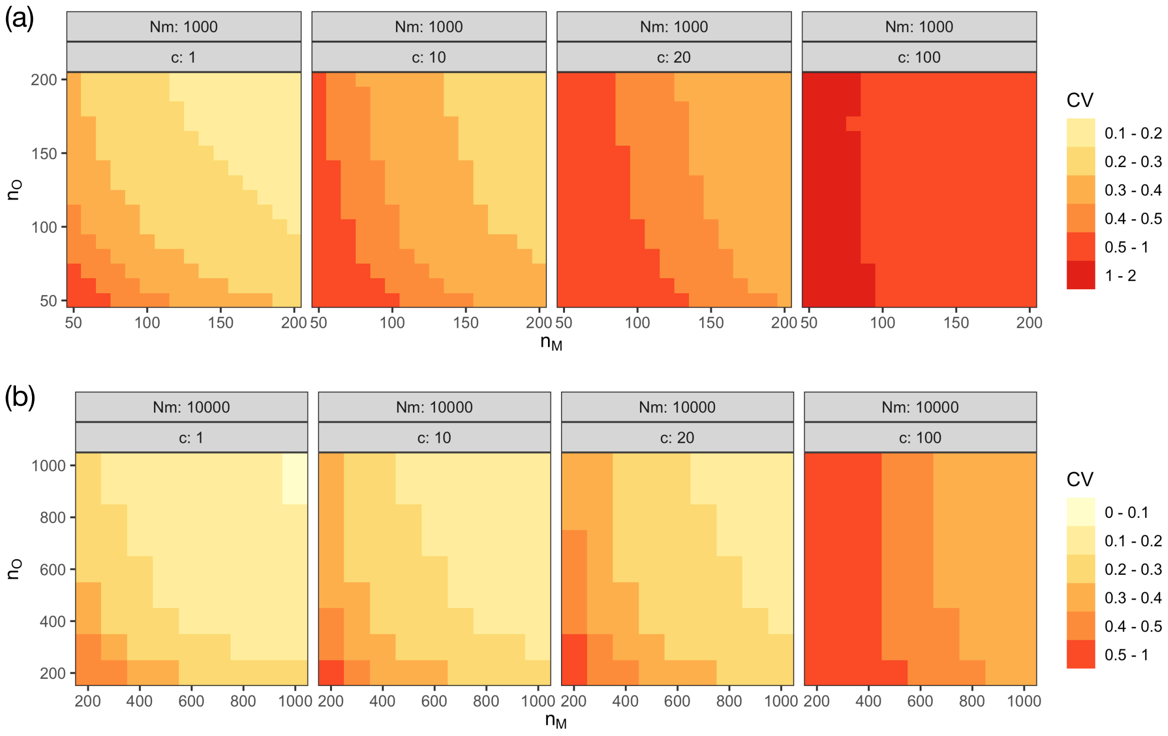

FIGURE S6 Heatmap showing the coefficient of variation of $\widehat{1/N_{\mathrm{m}}}$ as a function of both n_{M} and n_{O}. The value of the coefficient of variation is indicated in the legend. The value of the combined effect of parental and nonparental variations increases from left to right ($c = 1, 10, 20$, and 100). (a) $N_{\mathrm{m}} = 1{,}000$, (b) $N_{\mathrm{m}} = 10{,}000$.
